# 3D Mapping of Bone Channel of Blood Supply to Femoral Head in Proximal Femur

**DOI:** 10.3389/fsurg.2022.852653

**Published:** 2022-04-28

**Authors:** Shenghui Wu, Kun Quan, Wei Wang, Yingqi Zhang, Jiong Mei

**Affiliations:** ^1^ Department of Orthopaedic Surgery, Shanghai Jiao Tong University Affiliated Sixth People's Hospital, Shanghai, China; ^2^ Department of Orthopaedic Surgery, The First Affiliated Hospital of Nanchang University, Jiangxi, China; ^3^ Department of Biomedical Engineering, The Hong Kong Polytechnic University, Hong Kong, China; ^4^ Department of Orthopedic Surgery, Tongji Hospital, Tongji University School of Medicine, Shanghai, China

**Keywords:** nutrient foramina, mapping, hip surgery, three dimension (3D), distribution

## Abstract

**Background:**

A detailed depiction of nutrient foramina is useful for defining guidelines and minimising iatrogenic damage during hip surgery. Therefore, this study aimed to define the location and frequency of nutrient foramina in the proximal femur using mapping techniques.

**Methods:**

One hundred dry human cadaveric proximal femurs, comprising 56 left and 44 right femurs, were scanned using a three-dimensional scanner, with scanning distance 200 mm, precision 0.01 mm, and measuring point 0.04 mm. The image resolution of 1,310,000 pixels was obtained. Digital imaging models were acquired from the proximal femur surface. All the nutrient foramina in each model were identified and marked. The nutrient foramina models were superimposed on one another and oriented to fit a standard template of the femur’s proximal aspect. Three-dimensional mapping in the proximal femur’s nutrient foramina was performed.

**Results:**

The nutrient foramina’s location and dense zones were identified. The dense zones were distributed along the vascular course and gaps between the muscle attachment sites. Eighteen dense zones were identified and found to be location-dependent. They were located in the central part of the fovea capitis femoris, subcapital and basicervical areas of the femoral neck, and muscle attachment gaps of the femoral trochanter.

**Conclusions:**

The terminal branch of the nutrient vessels entering the nutrient foramina is at risk for iatrogenic damage during hip surgeries, especially in cases of close bone exposures. There are 18 dense zones that need to be considered for a safer approach to the proximal femur. To minimise iatrogenic damage to the nutrient vessels entering the nutrient foramina, the dense areas should be avoided when technically possible.

## Introduction

The nutrient foramen is a principal entry pathway for nutrient vessels, which are necessary for fracture healing or integration of implanted prostheses. The blood supply to the femoral head (FH) is derived primarily from the lateral femoral circumflex artery (LFCA) and the medial femoral circumflex artery (MFCA), which is a major contributor (1, 2). The precise anatomical location of the terminal branches of the MFCA has been well described using an imaginary clock face (3). The anatomical location of these arteries makes them vulnerable to injury either by trauma or iatrogenic damage during surgical interventions (4). Loss of the MFCA’s branches increases the risk of avascular necrosis (AVN) of the FH (5). Moreover, the blood vessels supplying the proximal femur enter through the nutrient foramina; thus, the positions of the nutrient vessels and foramina have considerable bearing on the safe surgical approaches to the proximal femur.

Previous studies have reported the location and number of nutrient foramina in various bones using macroscopic evaluation (6–10) and three-dimensional (3D) computed tomography (CT) measurement (11, 12). Because the bone surface is irregular, there is still no uniform location for the nutrient foramina in the proximal femur. Although nutrient vessels injected with marker substance can be used to map vessels and dissection allows for easier identification of nutrient vessels (3, 5), not all nutrient foramina can be shown owing to the soft-tissue occlusion and difference in sizes (8, 13). The 3D mapping technique combined with 3D bone reconstruction and fracture reduction can elucidate fracture lines and enhance understanding of the fracture (14). Moreover, an analysis of the overlap of all nutrient foramina on a standard template allowed for production of a 3-D nutrient foramina map to better grasp the nutrient foramina’s spatial distribution in the proximal femur.

This study aimed to define the location and frequency of nutrient foramina in the proximal femur. We hypothesised that there are dense nutrient foramina zones in the proximal femur.

## Methods

A total of 171 unpaired dry specimens of adult femurs were obtained from the Department of Anatomy to study the nutrient foramina’s 3D location and distribution in the proximal femur. General information regarding these specimens was unknown. Specimens were excluded based on the following criteria: (1) significant osteoarthritis or morphological changes within the FH (*N* = 20); (2) the presence of the epiphyseal growth plate of the FH (*N* = 8); and (3) damaged proximal femur (*N* = 43). One hundred dry femur specimens comprising 56 left and 44 right femurs were included.

The femur’s proximal surfaces were scanned using a 3D scanner (15) (Shanghai Digital Manufacturing Corp, Ltd, Shanghai, China), with scanning distance 200 mm, precision 0.01 mm, and measuring point 0.04 mm. The image resolution of 1,310,000 pixels was obtained. Digital imaging data were recorded from the proximal femur surface. The data were processed using 3-Matic software (Materialise), and each 3D model was rotated, normalised, and horizontally flipped where necessary, to best match the 3D standard model of the proximal aspect of the femur. The position of the nutrient foramen in the 3D models was defined by the centre of the nutrient foramen. The identifications and marks of the nutrient foramina were made while comparing with the corresponding specimens to confirm that no nutrient foramina were missed. Two observers with extensive experience of human anatomy performed each marking. Quality assessment was performed by two observers with adjudication by a third with more than 20 years of experience, until a consensus was reached.

The nutrient foramina of all specimens were represented in the standard model to obtain the distribution map of the nutrient foramina. The locations of the nutrient foramina in all the specimens were superimposed to depict the distribution of the nutrient foramina locations. The nutrient foramen area was defined as the area within 5 mm from the nutrient foramen. The closer the distance to the nutrient foramen, the higher the weight assigned to the nutrient foramen. Thus, all nutrient foramina of the specimens were superimposed to calculate the nutrient foramina frequency in the 3D model (16).

To clearly describe the surface morphology of the nutrient foramina, dense zones were identified based on osseous landmarks and peripheral vessels. The femoral neck surfaces in relation to peripheral vessels and the distribution of muscle attachment points in the femoral trochanter were illustrated by two authors. These spatial locations were determined based on the data of footprint areas, morphology of each muscle attachment, and courses of the LFCA and MFCA together with related studies (3, 8, 15, 17–20).

## Results

**Figure 1:**
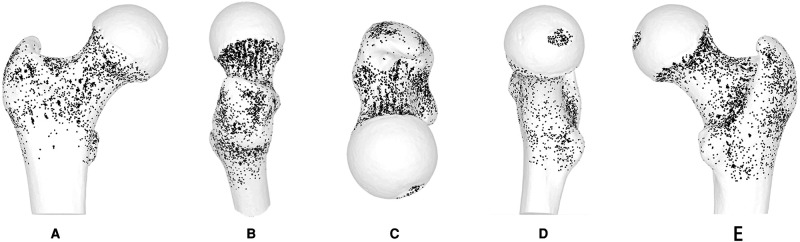
Representative views of the 3-D map of 100 specimens of all nutrient foramina in the proximal femur, including anterior, lateral, superior, medial and posterior views. Nutrient foramina are represented by points in the bone surface of the proximal femur.

**Figure 2:**
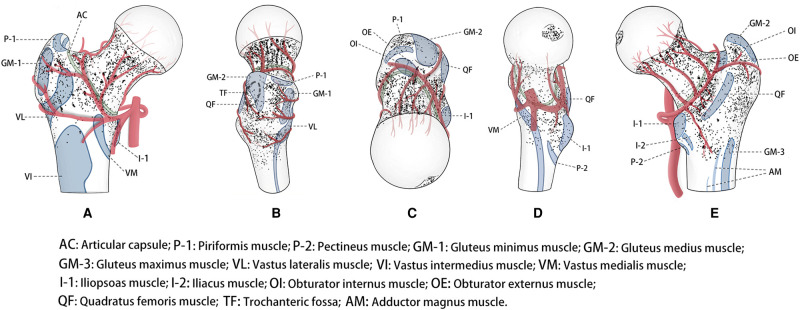
Representative views of location-dependent distribution of the nutrient foramina in the proximal femur with anatomical landmarks, including anterior, lateral, superior, medial and posterior views. The nutrient foramina (Points) clustered, suggested dense zones.

**Figure 3:**
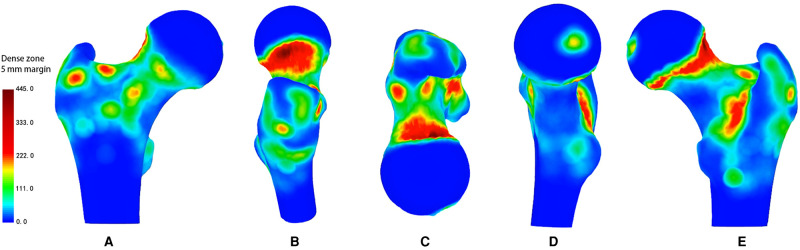
Representative views of the 3-D heat mapping with a 5-mm margin of 100 specimens of all nutrient foramina in the proximal femur, including anterior, lateral, superior, medial and posterior views. Red colour regions, defined as dense zones, represent higher frequency of nutrient foramina, while yellow and green colour regions, defined as lower dense zones, represent lower frequency of nutrient foramina.

Of 171 specimens, 71 were excluded. The nutrient foramina of the proximal femur of all the 100 specimens were visualised in a standard template (**Figure 1**). The nutrient foramina were densely located in the specimens with the distribution of the nutrient foramina being approximately parallel to the course of the nutrient vessels. The nutrient foramina in the femoral trochanter regions were mainly located in gaps between the muscle attachment sites (**Figure 2**). The 18 dense zones (DZs) identified were location-dependent (**Figures 1–3**). See also Videos 1, 2 in the supplementary material attached.

### Fovea Capitis Femoris

The DZ was located in the centre of the fovea capitis femoris (**Figures 1–3D**). Meanwhile, there was a reduction in the density of nutrient foramina from the central to peripheral regions.

### Femoral Neck

#### Anterior Region

Two DZs were distributed around regions of vessel bifurcation and channels through which the terminal branches of the anterior retinacular arteries enter the FH (**Figures 1–3A**).

#### Superior Region

There were three DZs. The first zone was located in the subcapital area of the femur’s neck, where superior retinacular arteries insert into the FH (**Figures 1–3B,E**). The nutrient foramina cluster varied from about 1/2 to 1/7 of the femoral neck length, toward the FH, showing a gradually decreasing trend from the anterior to posterior regions (**Figure 3B,E**). Two zones were located at the two sides of the basicervical femoral neck (**Figures 1–3E**).

#### Inferior Region

The DZ was located, about 1/7 of the femoral neck length, in the half posterior regions of the transcervical femoral neck through which the inferior retinacular arteries entered the FH (**Figures 1–3E**).

### Femoral Trochanter

#### Anterior Region

There were two DZs (**Figures. 1–3-A**). The first zone was situated in the medial-inferior regions of the attachment site of the gluteus minimus (GM-1), lateral regions of the attachment site of the articular capsule (AC), and superior regions of the proximal attachment site of the vastus lateralis (VL). The second zone was located in the medial-inferior regions of the attachment site of the VL, lateral regions of the attachment site of the AC, and superior regions of the proximal attachment site of the vastus intermedius and vastus medialis (VM).

#### Lateral Region

There was one DZ and two lower DZs (**Figures 1–3B**). The DZ was located in the regions below the distal attachment site of the gluteus medius (GM-2). The first lower DZ was located between the anterior regions of the attachment site of the GM-2 and the posterior regions of the attachment site of the GM-1. The second lower DZ was located between the posterior and superior regions of the attachment site of the VL and the inferior regions of the attachment site of the GM-1 and GM-2.

#### Medial Region

There were two lower DZs. The first zone was located in the anterior part of the lesser trochanter, which was predominately distributed among the inferior attachment site of the AC, anterior attachment site of the iliopsoas, and posterior attachment site of the VM (**Figures 1–3D**). The second zone was located in the trochanteric fossa’s central part (**Figure 2B**).

#### Posterior Region

There was one DZ and three lower DZs (**Figures 1–3E**). The DZ was located along the pertrochanteric crest and medial part of the attachment site of the quadratus femoris (QF). The first lower DZ was located below the attachment site of the GM-2 and above the attachment site of the QF. The second lower DZ was located in the lateral attachment site of the QF and above the proximal attachment site of the gluteus maximus. The third lower DZ was located at the proximal-medial attachment site of the adductor magnus and distal-lateral attachment site of the iliacus and pectineus.

## Discussion

Our results showed that there were 18 dense nutrient foramina zones in the proximal femur, confirming our hypothesis. We used the nutrient foramina mapping technique inspired by Armitage et al. (21) to analyse 100 dry specimens of adult femurs to improve our understanding of the distribution of the nutrient foramina in the proximal femur. We used qualitative evaluation based on previous quantitative anatomical studies (8, 13) to show the presence of dense nutrient foramina zones in the proximal femur. The DZs were parallel to the course of the nutrient vessels and were specifically located in the gaps between the attachment sites for muscles in the femoral trochanter. The 3-D map provided a more intuitive description of the morphology of the nutrient foramina in the proximal femur; thus, defining the DZs during hip surgery.

Detailed insights into the position of the nutrient foramina in the long bones can be useful in surgeries and medicolegal cases (7, 22). The blood supply is usually preserved in free vascularised bone grafts with living osteocytes and osteoblasts, while bone healing may be jeopardised by a low vascular network in and around bone grafts (23). Hence, the topographical knowledge of nutrient foramina is vital for specific surgical procedures to avoid causing damage to the circulation and protect the blood supply of the FH (3, 5, 7, 13, 24, 25).

Greater awareness of the nutrient foramina’s morphology can significantly affect the successful planning of hip surgeries. There have been very few studies on the effects of the surgical approach in hip surgeries on the impairment of blood supply to the proximal femur through the nutrient foramina. Previously, distribution of the nutrient foramina around the FH and neck junction was roughly described using a clock system that was not accurate enough for use in the clinical setting (13). Herein, the 3D analysis of the nutrient foramina on the surface topography of bones showed that the proximal femur had a substantial number of DZs. To protect the blood supply of the proximal femur, the anatomy of the LFCA and MFCA, together with associated terminal branches have been widely investigated (3, 26). However, these studies disregarded information regarding the vessels found on the proximal femur’s surface, a gap that has been filled in the present study. Our study showed that the DZs on the surface of the proximal femur were associated with regions covered by the nutrient vessels, and were located in the gaps between the attachment sites of muscles. The superior retinacular artery of the MFCA is the major blood supply to the FH (1–3, 27). When the first DZ of the superior region of the femoral neck provided by the SRA (2, 3) was damaged, there was a high risk of AVN, and the remaining zones needed care and caution. This may help surgeons form a mental map of the localisation of the nutrient foramina associated with the surgical area during osteotomies of the proximal femur (28–30).

Previous studies on the blood supply of the proximal femur focused on the circulation of extracapsular blood vessels. However, the nutrient foramina as the entrance and exit of a bone’s blood vessel is also the last way to protect the blood supply of the FH during hip surgery. Therefore, dense nutrient foramina zones need attention. The choice of surgical approaches to the proximal femur depends on the surgical goal and surgeon preference (31–33). In addition to the MFCA and LFCA, protection of the intracapsular vessels that pass through the pooled region of the retinacular vessels and the intracapsular dense nutrient foramina zone is also important for any approach. Generally, the anterior approach to the hip has less influence on the blood supply to the FH than the posterior approach, which was also confirmed by the description of the dense nutrient foramina zone therein. Besides, the joint capsule incision site and surgical exposure suggested by the Ganz approach minimised damage to the blood supply of the FH (5, 34). Although trochanteric osteotomy via the Ganz approach may cause iatrogenic damage to the dense zone of the posterior region in the femoral trochanter, it may have little impact on the blood supply to the FH.

There have been several studies on the blood supply to the hip aimed at improving the outcomes for hip procedures (17). However, the precise vascular anatomy of nutrient foramina in the proximal femur is not well defined. The regions covered by the retinacula of Weitbrecht in the femoral neck are associated with dense distribution of nutrient foramina (8). Yet, no qualitative information exists on the exact location and distribution of nutrient foramina in the proximal femur. Several studies have utilised quantitative or semi-quantitative measurements to investigate the morphologic and topographic properties of the nutrient foramina (6–13). However, the topographical description of the nutrient foramina has remained less intuitive because of the lack of direct methods to visualise them. Besides, 3D mapping techniques are currently being used to visualise articular injuries to better understand fracture patterns (35). CT is the standard tool for mapping studies. Nevertheless, this approach does not apply to the nutrient foramina since the precision of CT is not sufficient for it. Three-dimensional scanning of the nutrient foramina has higher accuracy and allows the storage of information on the surface of the bone, which can be repeatedly studied; thus, reducing the demand for bone specimens (15).

To our knowledge, this is the first study to elucidate the location of nutrient foramina of the proximal femur by utilising 3D mapping techniques based on 3D image data. The 3D mapping techniques have been widely used in studies to describe the morphology of various bone fractures (14, 16). The techniques used have evolved from CT-based bone reposition to 3D reconstruction using CT data (14). However, current CT scanners used in clinical practice do not meet the criteria for CT reconstruction to observe nutrient foramina (8). Currently, a 3D scanner can be applied to precisely measure the cross-sectional area of the nutrient foramina in the femoral neck of dry adult femur specimens (15). In our study, the 3-D model images obtained from a 3D scanner allowed for precise observation of the spatial locations of the nutrient foramina. Meanwhile, the nutrient foramina maps of the proximal femur illustrated the surface anatomy of spatial locations of the nutrient foramina, which could be explored to design the optimal surgical approach with the best exposure and minimise the risk of injury of crucial structures. Although the number and distribution of the nutrient foramina can be determined through painstaking macroscopic analysis. The 3D maps illustrated the anatomical location and surface area. Combined with high-definition images of the nutrient foramina recorded from a 3D scanner, the 3D maps can enhance the completeness and accuracy of our understanding of the nutrient foramina in the proximal femur.

There were some limitations to this study. First, this was a descriptive macrostructural study on the map of nutrient foramina in the proximal femur; thus, the map of microstructures, such as trans-cortical vessels (36), needs to be further studied. Second, the baseline characteristics of the specimens were not available for analysis. However, the site of the nutrient foramina has no significant correlation with the known bone age (37). During a period of rapid growth, the relative position of the femoral nutrient foramina in rats remains constant (38). Few studies have evaluated the correlation between the baseline characteristics and location of the foramina in the proximal femur.

In conclusion, the nutrient vessels entering the nutrient foramina are at risk of iatrogenic damage during hip surgery, especially when soft-tissue exposure is close to the course of the vessels and gaps between the muscle attachments. Eighteen DZs for iatrogenic vessel injury were identified: one was located in the central part of the fovea capitis femoris; six were located in the subcapital and basicervical areas of the femoral neck; and 11 were located in the muscle attachment gaps of the femoral trochanter. To minimise iatrogenic damage to nutrient vessels entering the nutrient foramina, the direction of soft-tissue exposure should be parallel to the course of vessels where technically possible.

## Data Availability

The raw data supporting the conclusions of this article will be made available by the authors, without undue reservation.
